# Prevalence of Keratoconus and Associated Risk Factors Among High School Students in Couva, Trinidad: A Cross-Sectional Study

**DOI:** 10.3390/vision9040089

**Published:** 2025-10-20

**Authors:** Ngozika Esther Ezinne, Shinead Phagoo, Ameera Roopnarinesingh, Michael Agyemang Kwarteng

**Affiliations:** 1 Optometry Unit, Department of Clinical Surgical Sciences, University of the West Indies, Saint Augustine Campus, Saint Augustine 330912, Trinidad and Tobago; shinead.phagoo@my.uwi.edu (S.P.); ameera.roopnarinesingh@my.uwi.edu (A.R.); kwartengmichaelagyemang@gmail.com (M.A.K.); 2 Bathurst Rural Clinical School, Western Sydney University, Bathurst 2795, Australia; 3 Translational Health Research Institute, Western Sydney University, Campbelltown 2560, Australia

**Keywords:** keratoconus, prevalence, high school children, risk factors, Couva, Trinidad and Tobago

## Abstract

**Purpose:** This study aimed to determine the prevalence and associated risk factors of keratoconus (KC) among high school students in Couva, Trinidad and Tobago. **Method:** A cross-sectional, school-based approach was used, involving a simple random sampling technique to select schools and students. A structured questionnaire assessed KC risk factors, while clinical assessments, including visual acuity, refraction, slit lamp biomicroscopy, and topography, were performed. Data were analyzed using R. Exact tests were used for KC (*n* = 2 cases) and robust Poisson regression estimated adjusted prevalence ratios for the ‘at-risk’ screening endpoint. **Results:** A total of 432 students aged 12–17 years participated, with a response rate of 97.5%. Most participants were of East Indian descent (48.1%), female (52.1%), and 14 years old (23.1%). Approximately 47.7% (95% CI 43.0–52.5%) were at risk of KC, with 0.5% (2/432; exact 95% CI 0.06–1.67%) diagnosed with the condition. The most common risk factors were eye rubbing (87.4%), over eight hours of sun exposure weekly (71.8%), and atopy (68.4%). KC was observed to be significantly higher among people with a family history (*p* = 0.018). **Conclusions:** The study highlights a low prevalence and a high risk of KC among high school students, with a strong link to family history and common risk factors such as eye rubbing and sun exposure. These findings emphasize the urgent need for regular KC screening in schools to ensure early diagnosis and effective management.

## 1. Introduction

Globally, corneal blindness is the third leading cause of blindness after cataracts and glaucoma, even though 80% of causes of corneal blindness are preventable or treatable [1]. One of the known causes of corneal blindness is keratoconus (KC), which was clinically defined more than a century ago. KC is an asymmetric ectatic corneal disorder characterized by progressive corneal thinning that results in corneal protrusion, irregular astigmatism, and reduced vision [2,3]. The disease usually develops in adolescence or early adulthood and progresses into the third or fourth decade of life [2,4]. It is one of the major causes of visual impairment with significant socio-economic impacts in the pediatric population [5]. The diverse associations of KC suggest that it results from several different pathological processes; it may therefore be regarded as a heterogeneous group of conditions with identical clinical signs but with a varied pathogenesis [6].

The cause of KC is still not known but there are risk factors which make individuals more susceptible to developing the disease. Studies have revealed that genetics, environmental factors, ethnicity, age, eye rubbing, atopy, family history, ultraviolet exposure, parental consanguinity, hormones, inflammatory response, and pregnancy are major risk factors for keratoconus [7].

The prevalence of KC varies with ethnicities, geographical locations, and methods of detection [2,3,5]. A higher prevalence was reported among individuals with a family history of KC, eye rubbing, and a history of parental consanguinity [2]. The prevalence of KC was recently reported to be 1 in every 350 people in the general population [7], with Asians and countries with hot weather conditions recording a higher prevalence [2,3,5,8,9]. A low prevalence of KC was reported in Europe [10]. The prevalence of KC in the pediatric population was 0.16% [11]. Prevalence figures of 1:200 and 1:25 were recorded among children in Lebanon [12] and Saudi Arabia [9], respectively. A study among school children in New Zealand reported a prevalence of 0.52% [13].

Keratoconus is typically diagnosed during adolescence; however, in younger children it may be easily misdiagnosed/missed, as it rarely causes amblyopia. Also, the disease course is more aggressive in children than in adults, and children are more likely to have advanced disease at presentation; therefore, early detection is optimal for better management [11]. Early detection of KC will allow for providing optimal treatment to reduce progression to more advanced stages of vision loss. Currently, there are limited data on keratoconus studies in adolescents globally. There are no published studies on KC in the Caribbean, especially in Trinidad and Tobago populations. However, anecdotal evidence suggests that there is a high presentation of the disease in ophthalmic services among adolescents in Trinidad and Tobago. The only available data is mainly from Caribbean populations living outside the Caribbean. It is important to know about the epidemiological of KC among high school students in Trinidad and Tobago to develop an appropriate management strategy for the condition. This study was conducted to determine the prevalence of KC and associated risk factors among high school children in Trinidad and Tobago. The results of this study can be used to develop guidelines for the diagnosis, prevention, and treatment of KC to reduce the burden associated with the disease.

## 2. Material and Methods

### 2.1. Study Design

This was a descriptive cross-sectional, school-based study conducted from September 2022 to April 2023.

### 2.2. Study Setting

The study was conducted in Couva, a town located in west-central Trinidad with a population of approximately 48,858 people [14]. The Couva Multi Training Facility (CMTF) is a popular landmark located in Couva. It is where the Couva Hospital and UWI Optometry Clinic are situated. The ethnic composition of the population in Couva is 34.2% African, 35.4% East Indian, 22.8% mixed and 7.6% other races [15,16]. The climate in Couva is tropical, with high relative humidity. Most residents are Christians (55.2%) followed by Hindus (18.2%) and others are Muslim (4.9%) or unknown (21.7%) [16].

There are six secondary schools in Couva, and all are near the CMTF. The enrolment rate in high school is 93.2% [14]. The age range of students in high schools is 12–16 years from Forms 1–5 [14]. The secondary schools in Couva are grouped according to 3 main categories: government-funded, partially government-funded, and private schools. Government-funded schools are multi-religious institutions and mixed-gender schools.

### 2.3. Sample Size and Study Population

High school students in Couva constituted the study population. The minimum sample size was calculated with the single-population proportion formula (finite-population correction), implemented in Raosoft:

n = N XX + N − 1,X = Z1 − α/22 p (1 − p)MOE2n \;=\; \frac{N\;X}{X + N − 1}, \qquad X \;=\; \frac{Z_{1 − \alpha/2}^{2}\;p\;(1 − p)}{\text{MOE}^{2}}n = X + N − 1NX,X = MOE2Z1 − α/22p(1 − p)

where nnn is the required sample size, NNN the population size, Z1 − α/2Z_{1 − \alpha/2}Z1 − α/2 the standard normal deviate at the chosen confidence level, ppp the expected proportion, and MOE the absolute margin of error.

We used a 95% confidence level (Z = 1.96) (Z = 1.96) (Z = 1.96), an absolute margin of error of 0.5 percentage points (MOE = 0.005), and an assumed population size of 100,000 students. The expected prevalence ppp was set to 0.29% (*p* = 0.0029) (*p* = 0.0029) (*p* = 0.0029), the mean of three published estimates: 0.52% (New Zealand), 0.19% (Norway), and 0.16% (USA). Substituting, X = 1.962 × 0.0029 × (1 − 0.0029)0.0052 ≈ 444, n = 100,000 × 444,444 + 100,000 − 1 ≈ 443.X = \frac{1.96^{2}\times 0.0029\times(1−0.0029)}{0.005^{2}}\approx 444,\quad n = \frac{100{,}000\times 444}{444 + 100{,}000−1}\approx \mathbf{443}.X = 0.00521.962 × 0.0029 × (1 − 0.0029)≈444,n = 444 + 100,000 − 1100,000 × 444 ≈ 443.

Accordingly, the target sample size was 443. Of these, 432 students provided consent and completed the questionnaire (response rate 97.5%).

### 2.4. Inclusion and Exclusion Criteria

High school students in Couva between the ages of 11 and 20 years who consented to participate in the study were included in the study. Students with corneal pathologies, trauma to the cornea, or any condition of the cornea unrelated to KC were excluded.

### 2.5. Sampling Technique

Three schools were selected from the six high schools in Couva based on their proximity to CMTF. The three schools were government-funded and admitted male and female students of different religious backgrounds. Simple random sampling was used to recruit participants from their schools using a picker wheel. A picker wheel is an interactive tool used to randomly select an option from a predefined list of choices. It often appears as a virtual spinning wheel, much like a prize wheel or game show wheel, where users can click a button to spin and have the wheel stop on a random selection. First Step: The sample size was divided by 3 to determine how many students would be selected to participate from each school. Thus, 148 students were selected from each school. Secondary schools in T&T have 5 grades, therefore 148 was divided by 5. A total of 30 students were selected from each grade. Second Stage: The names of all the students in each form were gathered from a list provided by the Form mistress/master. Their names were coded and inserted into a software called Picker Wheel (Version 96; PickerWheel.com). This software allows for the use of simple random sampling to select each student. The wheel was spun 30 times to select 30 students from the Form. This was done for each grade in each school to get all 443 participants needed for the study. After, 443 participants were selected, and the consent forms were given out. Those whose consent was obtained were invited to participate in the study. A face-to-face interview was conducted using a structured questionnaire to assess the risk factors of KC.

### 2.6. Data Collection Tools

Keratoconus risk assessment questionnaire, visual acuity chart, autorefractor, retinoscope, slit lamp, and topographer were used in the data collection.

### 2.7. Data Collection Procedure

After obtaining ethical approval and the relevant permissions from the Ministry of Education, selected schools’ principals were visited, and information about the study and the reason to participate in the study was explained. A request to allow their students to participate in the study was made and a meeting was scheduled with the students to explain the purpose of the study and the reason to participate in the study. Information document and consent forms were shared with selected students for their parents. A day was scheduled for data collection in their various schools. Those whose consent to participate in the study was obtained were invited to participate in the study. Participants completed a structured, interviewer-administered KC risk questionnaire (Supplementary Materials) adapted from prior literature [6,9,11,12,13]. All items were captured as dichotomous responses (Yes/No). Responses were coded Yes = 1/No = 0.

Each school provided a room where the assessments were done to avoid disrupting their normal school activities. Students suspected of having KC during the study were counseled and referred to the optometry unit of the University of the West Indies at CMTF or the nearest eye clinics or hospitals to their homes for management. Three optometrists were recruited to assist with the data collection including administering the questionnaire and conducting the clinical assessments.

The three optometrists were trained before the data collection. The roles were shared among the research assistants in such a way that one person performed the same role throughout the data collection to avoid inter-examiner variability. Before the initiation of the study, all field investigators were familiarized with the standard operating procedures involved. A pilot was conducted on 50 students outside the selected schools to validate the data collection forms to minimize inter-observer variation.

### 2.8. Clinical Assessment

Demographic information of the participants including student’s age, gender, and grade was documented at the time of data collection. The information was verified before examination with the help of the class supervisors. A unique code number was given to each participant. Each participant was given the questionnaire to complete and to determine their risk profile. Distance visual acuity was measured with a retro-illuminated log MAR chart with five optotypes on each line (Precision Vision, Villa Park, IL, USA). Visual acuity measurements were taken at a distance (4 m) and near (30 cm or habitual reading distance). The EDTRS chart was used to measure distance visual acuity, and the reduced ETDRS chart was used to measure near visual acuity. Retinoscope was used to check for the presence of a scissor reflex when scanning the cornea. The curvature and dioptric power of the anterior surface of the cornea were measured using a handheld autokeratorefractor (Retinomax K-Plus; Nikon, Tokyo, Japan) according to the manufacturer instruction manual. The handheld autorefractor was used to determine the refractive status, corneal curvature, and dioptric power. A handheld slit lamp was used to assess the anterior segment. All instruments were calibrated daily and a minimum of five readings with valid confidence rankings were obtained for each eye and average was taken. A topographer (Topcon CA-800, Tokyo, Japan) was used to assess the cornea curvature, and a final diagnosis of KC was made by the principal investigator. Those found to have KC were counseled and referred to the CMTF. A KC survey risk questionnaire was used to assess risk factors of KC including eye rubbing, eczema, asthma, contact lens wear, refractive error (astigmatism), frequency of changing spectacles, family history of KC, UV/sun exposure, frequency of near work activity, history of laser surgery, consanguinity, and associated syndromes like Down, Ehlers–Danlos, or Marfan syndromes.

Clinical measurements and quality control.

Autorefraction and keratometry: Retinomax K-Plus; ≥5 readings/eye; averaged if device confidence acceptable (per manufacturer).

Topography: Topcon CA-800 4-map report; only scans with manufacturer “OK” quality flag were analyzed.

Illumination: routine room lighting; illuminance not standardized.

### 2.9. Outcome Variables

Primary outcome: KC prevalence with exact (Clopper–Pearson) 95% CI. Secondary outcome: prevalence of the “at-risk” screening category; distribution of keratometric/topographic parameters.

### 2.10. Criteria Used to Determine the Risk Factors of KC

Eye rubbing was defined as vigorous self-reported rubbing of the eye with the knuckle, rated with an intensity of 3 or higher on a 5-point scale. Parental consanguinity referred to instances where both parents are either first or second cousins or are related by blood within a specific degree of kinship. Sunlight exposure was defined as at least eight hours of cumulative natural sunlight exposure per week. Near work involved tasks requiring sustained close visual attention (e.g., reading, using a mobile phone, or working on a computer) for more than eight hours per day. A positive family history of KC was defined as having at least one relative with a confirmed diagnosis of the condition. Atopy was characterized by self-reported allergies, including eczema, asthma, hay fever, food allergies, and other allergic conditions such as pollen, dust, animal fur, and vernal keratoconjunctivitis.

Additionally, a visual acuity (VA) of 6/12 (LogMAR 0.3) or worse in at least one eye, which can be improved with glasses, was considered a relevant factor. Myopia was defined as a refractive error of −0.5 diopters (D) or less, while astigmatism was defined as a minus cylinder of −0.75 D or less. Individuals with at least three risk factors, including one established factor, were referred for further clinical evaluation, which included retinoscopy, autorefraction, slit-lamp biomicroscopy, topography, and optical coherence tomography (OCT). The diagnosis of KC was based on clinical signs such as a scissor reflex, irregular astigmatism during retinoscopy, topography, and OCT results, as well as slit-lamp observations, including Vogt’s striae, Fleischer rings, and Munson’s sign.

Participant classification.

KC: diagnosis required ≥1 clinical sign (e.g., Vogt’s striae, Fleischer ring, scissoring reflex) and abnormal topography on Topcon CA-800 meeting either of the following a priori criteria: Kmax ≥ 47.2 D or asymmetric bow-tie with skewed radial axes and inferior–superior (I–S) asymmetry ≥ 1.4 D. Staging used Amsler–Krumeich.

KC—suspect: abnormal topographic indices suggestive of ectasia (e.g., localized inferior steepening or I–S 0.8–1.39 D) without meeting KC criteria and no slit-lamp signs.

No KC: neither clinical signs nor abnormal topography.

Screening risk category (“at-risk”): based on questionnaire and basic screening results, participants were classified “at-risk” if they had ≥3 risk factors including ≥1 established factor.

Established factors (Yes/No): eye rubbing rated ≥3/5; atopy (eczema/asthma/allergic rhinitis/VKC); family history of KC in a first-degree relative; parental consanguinity; and ≥8 h/week sunlight exposure.

Other factors (Yes/No): ≥24 h/week near work; refractive error present; and spectacles changed ≥1×/year.

All questionnaire items were interviewer-administered and dichotomous (Yes = 1/No = 0).

### 2.11. Data Analysis

Descriptive statistics (n, %, and mean ± SD) summarized all variables. KC prevalence was calculated as a proportion with exact (Clopper–Pearson) 95% CIs. Because only two KC cases were observed, between-group comparisons used Fisher’s exact tests. Where effect sizes were informative, median-unbiased exact odds ratios with exact 95% CIs from conditional logistic models were reported. For the secondary “at-risk” screening endpoint, associations with risk factors were estimated as prevalence ratios (PRs) using Poisson regression with robust variance, adjusted for age (years) and sex. Analyses were conducted in R (version [4.3.2]); two-sided α = 0.05.

## 3. Results

### 3.1. Demographic Profile of Participants

A total of 432 students aged 12–17 years with a mean age of 14.08 (± 1.49) years participated in the study giving a 97.5% response rate. The majority were of East Indian descent (48.1%), females (52.1%), 14 years old (23.1%) and in Form 2 (25.5%) (Table 1).

### 3.2. Clinical Characteristics of Study Participants

Most of the participants had VA better than 6/12 on the right (87.0%) and left eye (88.9%). A significant proportion (32.9%) had refractive error, and myopia was the most prevalent refractive error among them (65%). About half (47.2%) were at risk of developing KC (Table 2).

### 3.3. Demographic Profile of Participants at Risk of Keratoconus

A total of 206 (47.7%, 95% CI 43.0–52.5%) participants were at risk of developing keratoconus. The majority of those at risk were female (54.4%), 15 years old (21.4%), and East Indian (47.6%). The most prevalent risk factor among them was eye rubbing (87.4%), followed by more than 8 h of exposure to sunlight per week (71.8%), and having atopy (68.4%) (Table 3).

### 3.4. Keratometric and Topographic Measurement of the Participants at Risk of KC

Participants at risk of KC had a maximum of −6.50 D, −7.50 D, and 66.5 D for myopia, astigmatism, and Kmax reading, respectively. The mean unaided and aided visual acuity of those with KC on both eyes were 1.33 Log MAR (6/120) and 0.35 Log MAR (approximately 6/12), respectively. The average cornea thickness of those with KC was 398 µm (Table 4).

### 3.5. Prevalence of KC

The prevalence of keratoconus was 0.5% (2/432; exact 95% CI 0.06–1.67%). One (50%) was East Indian (50%) and the other was mixed (50%). They were aged 14 (50%) to 17 (50%) years with a mean (±SD) age of 15.5 (±2.12) years. Additionally, one (50%) was female and one (50%) was male. The at-risk screening prevalence was 47.7% (206/432; 95% CI 43.0–52.5%). They had localized corneal thinning, Vogt’s striae, and Fleishjer’s ring. Both had advanced stages of KC according to Amsler–Krumeich classification [17] and were unaware of having KC before they participated in the study.

### 3.6. Comparison of Risk Factors for Participants with and Without KC

Both participants with KC had refractive error, atopic diseases, eye rubbing, and no parental consanguinity. Half (50%) of the participants with KC had a family history, spend ≥8 h of sunlight exposure, and change their glasses at least once a year (Table 5).

### 3.7. Correlation of KC with the Variables

With only two KC cases, comparative analyses relied on exact methods. Family history was more frequent in KC (1/2) than non-KC (3/430) (Fisher *p* = 0.018). The exact OR was 71.1 (exact 95% CI 2.8–∞)). No other risk factor differed on Fisher’s exact testing (all *p* > 0.10) (Table 6).

## 4. Discussion

To the best of our knowledge, this study represents the first attempt to survey and assess the prevalence of keratoconus (KC) among high school students in the Caribbean, specifically in Trinidad and Tobago. Our findings revealed that approximately half of the children who participated in the study were at risk of developing KC, with two of them diagnosed with the condition. Eye rubbing emerged as the most prevalent established risk factor for KC, followed by exposure to sunlight and a history of atopy. The majority of those at risk were females, East Indians, and individuals aged 15 years. Participants diagnosed with KC exhibited refractive errors, eye rubbing behaviors, atopic diseases, advanced stages of KC, and were unaware of their condition. The odds of developing KC were notably higher among participants with a family history of the condition.

The prevalence of participants at risk of developing KC in this study indicates a concerning proportion of children who may be susceptible to the condition. The proportion at risk in our study was notably higher than the 34.5% reported in a study conducted in Cameroon [18]. Additionally, the prevalence of KC in our sample, though not extensive in terms of total number of diagnosed cases, suggests a significant potential for undiagnosed cases of KC among children in Trinidad and Tobago. This highlights the need for further longitudinal monitoring and assessment, particularly using tools such as Pentacam to detect early-stage KC that may have been missed by the methods employed in this study. We recommend further population-based studies to assess the broader trends in KC prevalence in Trinidad and Tobago, to facilitate more comprehensive comparisons with our findings.

The prevalence of KC observed in our study (0.5%) aligns closely with similar findings from studies in New Zealand [13] and Norway [4], where rates of 0.5% and 0.19% were reported, respectively. However, it is lower than the 1.5%, 4.79%, and 4.4% rates reported in studies from Palestine [5], Saudi Arabia [9], and Lebanon [19]. The use of a topographer in our study as opposed to a Pentacam or tomographer—common in other studies—could explain these variations, as a topographer only assesses the anterior surface of the cornea, while a Pentacam evaluates both the anterior and posterior surfaces, making it more sensitive for detecting KC. Also, differences in methodology, and environmental and geographical location could also be another factor. For example, our sample size was smaller than the sample size used in the study in Saudi Arabia. In addition, past studies showed that the prevalence of KC is highest among individuals from the Middle East and North Africa.

Eye rubbing, identified as the most common risk factor in our study, may be attributed to the dusty environment and the prevalence of pollen-laden air that is typical of Trinidad and Tobago, which can provoke allergic reactions and frequent eye rubbing. Similar findings have been reported in studies from the Middle East [5,9,20,21,22,23]. Although eye rubbing was the most frequently reported risk factor, no statistically significant association was found between eye rubbing and KC in our study, contrary to findings from previous research [24]. This discrepancy could be due to differences in how eye rubbing is quantified, with most studies focusing solely on the frequency of rubbing rather than factors such as the force, method, duration, or seasonal variations. Additionally, environmental factors like high dust levels in certain climates may lead to more frequent eye rubbing, both among individuals with and without KC, thereby masking potential associations.

Family history has consistently been linked to a higher risk of developing KC in numerous studies [25,26,27] and our findings align with this established connection. The association of KC among participants with a family history reported in the current study is consistent with previous studies from Iraq [20,28].

The three primary risk factors identified in our study—eye rubbing, sunlight exposure, and atopy—while not statistically significant in association with KC, are similar to those reported in studies from Iraq and Iran [28,29]. Interestingly, the higher prevalence of females among those at risk of developing KC observed in our study aligns with findings from prior research [30], although the association between KC and gender remains inconclusive and warrants further investigation. There is a clear need for additional studies to elucidate potential gender-related factors in the development of KC.

The gender distribution of KC in our study, which was found to be equal, contradicts findings from other studies in Iran [31] and Mexico [30]. However, the relatively small sample size of our study limits its ability to generalize findings to the broader population, and we recommend future research with larger sample sizes to explore gender distribution more comprehensively.

The equal gender distribution of keratoconus recorded in our study is contrary to findings from other studies in Iran [31] and Mexico [30]. However, the sample size used in our study is not enough to generalize to the entire population. There is a need to conduct more studies on KC in Trinidad with a large sample size to compare gender distribution.

The mean age of participants diagnosed with KC in our study (16.1 years) was similar to the ages reported in studies from Mexico [30] and New Zealand [13], further reinforcing the notion that KC typically manifests during adolescence. This suggests the importance of implementing early screening programs for KC in high-risk populations, such as children and adolescents in Trinidad and Tobago.

It is noteworthy that both participants with diagnosed KC in our study had advanced stages of the disease, which contrasts with the earlier stages commonly observed in studies from New Zealand [13]. The use of a topographer in our study, which has limited sensitivity compared to a Pentacam or tomographer, may account for this discrepancy. Therefore, we strongly recommend utilizing more advanced diagnostic tools such as Pentacam for early detection of KC in pediatric populations in Trinidad and Tobago.

## 5. Strength and Limitations

The findings of this study should be interpreted with caution, as they are based solely on a sample of children from secondary schools in Couva, Trinidad and Tobago. Given the small sample size, the study’s ability to identify additional associated risk factors was limited. Furthermore, the use of a topographer, rather than a Pentacam, restricted our capacity to detect early-stage keratoconus (KC). Additionally, the study’s reliance on self-reported data from participants introduces potential bias in terms of honesty and responsiveness to the questionnaires (especially on family history). The use of a topographer for children identified as at risk of KC may have further influenced the reported prevalence, as children with early or asymptomatic KC could have been missed. Moreover, since our study was conducted in an institutional setting, it is not representative of the broader population. Despite these limitations, the findings from our study are consistent with those from similar research in other populations. The sample size, although small, was sufficient to provide valuable insights into the prevalence and risk factors of KC within the study area. The random selection of participants ensured diversity, with representation from all major ethnic groups, and the high response rate further bolstered the reliability of our findings. Notably, the fact that 100% of the participants diagnosed with KC were newly identified underscores the importance of early screening in school children, highlighting a significant gap in the current diagnostic and management practices in Trinidad and Tobago. Also, ambient illumination was not standardized or recorded, which may influence pupil size and accommodation and thus introduce additional variability in non-cycloplegic handheld autorefraction/keratometry. Future studies will standardize and document room illuminance with a lux meter and consider cycloplegia where feasible. A more comprehensive, population-based study is therefore recommended to better assess the prevalence and risk factors for KC in Trinidad and Tobago.

## 6. Conclusions

The prevalence of KC among high school students in Trinidad and Tobago is low but with a high risk. KC was associated with a positive family history. Key risk factors identified include eye rubbing, prolonged sun exposure (exceeding eight hours), and the presence of atopic diseases. These findings emphasize the urgent need for regular KC screening in high school students across the country to facilitate early detection and appropriate management. When exposure to irritants and airborne pollen is unavoidable, use of lubricating eye drops or artificial tears to soothe and protect the ocular surface, along with cooling compresses to relieve itching, is recommended. This study highlights the significant risk of underdiagnosis of KC in children and adolescents in Trinidad and Tobago. To gain a deeper understanding of the condition’s prevalence, risk factors, and early markers, future research should employ advanced diagnostic tools, such as Pentacam, and include larger, more representative sample sizes. Such efforts are essential for informing preventive strategies and improving the management of KC within the population.

## Figures and Tables

**Table 1:** Demographic profile of participants.

Variable	Frequency (N = 432)	Percentage (N = 100%)
Gender		
Female	225	52.1
Male	207	47.9
Age		
12	72	16.7
13	97	22.5
14	100	23.1
15	79	18.3
16	53	12.3
17	31	7.2
Ethnicity		
East Indian	208	48.1
African	96	22.2
Chinese	11	2.5
Mixed	117	27.1
Form Class		
1	107	24.8
2	110	25.5
3	85	19.7
4	67	15.5
5	63	14.6

**Table 2:** Clinical characteristics of participants.

Variables	Frequency (N = 432)	Percentage (N = 100%)
Visual Acuity		
Right Eye Unaided VA		
Better than 6/12	376	87.0
6/12 or worse	56	13.0
Left Eye Unaided VA		
Better than 6/12	384	88.9
6/12 or worse	48	11.1
Both Eyes Unaided VA		
Better than 6/12	386	89.3
6/12 or worse	46	10.7
Refractive Error		
Yes	142	32.9
No	290	67.1
Type of Refractive Error		
Myopia	92	65
Astigmatism	36	25
Hyperopia	14	10
At Risk of KC		
Yes	206	47.1
No	226	52.9

**Table 3:** Demographic profile of participants at risk of keratoconus.

Variable	Frequency (N = 206)	Percentage (N = 100%)
Gender		
Female	112	54.4
Male	94	45.6
Age		
12	39	18.9
13	42	20.4
14	38	18.4
15	44	21.4
16	28	13.6
17	15	7.3
Ethnicity		
East Indian	98	47.6
African	45	21.8
Chinese	6	2.9
Mixed	57	27.7
Refractive Error		
Yes	106	51.5
No	100	48.5
Eye Rubbing ≥ 3/5		
Yes	180	87.4
No	26	12.6
Family History of Keratoconus		
Yes	3	1.5
No	203	98.5
Parental Consanguinity		
Yes	29	14.1
No	177	85.9
Presence of Atopic Diseases		
Yes	141	68.4
No	65	31.6
≥8 h Sunlight Exposure per Week		
Yes	148	71.8
No	58	28.2
≥24 h Near Work per Week		
Yes	95	46.1
No	111	53.9
Frequency of Changing Spectacles		
Once a year or more	43	20.9
Less than once a year/No spectacles	163	79.1

**Table 4:** Keratometric and topographic measurements of the participants.

	Min	Max	Mean	STD
Sphere (DS)	−0.50	−6.50	−4.44	2.21
Cylinder (DC)	−0.50	−7.50	−5.19	1.82
Kmax (D) without KC	40.25	42.50	41.38	1.13
Kmax (D) with KC	50.18	66.5	58.34	8.16

Note: Summaries are participant-level using the worse eye (higher Kmax). STD = Standard deviation.

**Table 5:** Comparison of risk factors in participants with and without KC.

	With Keratoconus N (%)	Without Keratoconus N (%)	Total = 432 (%)
Refractive Error			
Yes	2 (0.5)	140 (32.4)	142 (32.9)
No	0 (0.0)	290 (67.1)	290 (67.1)
Eye Rubbing ≥ 3/5			
Yes	2 (0.5)	303 (70.1)	305 (70.6)
No	0 (0.0)	127 (29.4)	127 (29.4)
Family History			
Yes	1 (0.2)	3 (0.7)	4 (0.9)
No	1 (0.2)	427 (98.8)	428 (99.1)
Atopic Disease(s)			
Yes	2 (0.5)	207 (47.9)	209 (48.4)
No	0 (0.0)	223 (51.6)	223 (51.6)
≥8 h sunlight exposure per week			
Yes	1 (0.2)	221 (51.2)	222 (51.4)
No	1 (0.2)	209 (48.4)	210 (48.6)
≥24 h of near work per week			
Yes	0 (0.0)	121 (28.0)	121 (28.0)
No	2 (0.5)	309 (71.5)	311 (72.0)
Frequency changing spectacles			
More than once a year	1 (0.2)	44 (10.2)	45 (10.4)
Less than once a year/no spectacles	1 (0.2)	386 (89.4)	387 (89.6)
Parental Consanguinity			
Yes	0 (0.0)	43 (10.0)	43 (10.0)
No	2 (0.5)	387 (89.6)	389 (90.0)

Values are n (%) at the participant level (N = 432).

**Table 6:** **Associations with keratoconus (KC): small-sample corrected odds ratios and Fisher’s exact tests**.

Risk Factor	KC Yes (n = 2)	KC No (n = 430)	OR (Haldane–Anscombe)	95% CI	Fisher’s Exact *p* (Two-Sided)
**Refractive error (yes vs. no)**	2/0	140/290	10.34	0.49–216.79	0.108
**Eye rubbing ≥ 3/5 (yes vs. no)**	2/0	303/127	2.10	0.10–44.06	1.000
**Family history (yes vs. no)**	1/1	3/427	122.14	6.27–2378.10	**0.018**
**Atopic disease (yes vs. no)**	2/0	207/223	5.39	0.27–107.64	0.233
**Sunlight ≥ 8 h/week (yes vs. no)**	1/1	221/209	0.95	0.06–14.48	1.000
**Near work ≥ 24 h/week (yes vs. no)**	0/2	121/309	0.51	0.03–8.23	1.000
**Change spectacles > 1×/year (yes vs. no)**	1/1	44/386	8.77	0.54–142.74	0.198
**Parental consanguinity (yes vs. no)**	0/2	43/387	1.78	0.08–37.72	1.000

Notes. ORs computed with the Haldane–Anscombe correction (add 0.5 to all cells when any cell is zero). CIs are Wald-type on the log(OR) with the same correction. *p*-values are Fisher’s exact (two-sided) based on fixed margins. Given **n = 2** KC events, estimates are highly imprecise; emphasis should be on the direction of effect and the exact *p*-values rather than the OR magnitude. Bolded figures signify statistically significant association.

## Data Availability

Due to patient privacy protection and ethical restrictions, the data presented in this study are available on request from the corresponding author.

## Supplementary Materials

The following supporting information can be downloaded at https://www.mdpi.com/article/10.3390/vision9040089/s1.
