# Supplementary material for: Prevalence of Keratoconus and Associated Risk Factors Among High School Students in Couva, Trinidad: A Cross-Sectional Study
Source: Vision (Basel). 2025 Oct 20;9(4):89. doi: 10.3390/vision9040089 (PMC12551074; doi:10.3390/vision9040089)
Supplement: Supplementary file 1 [file vision-09-00089-s001.zip › vision-3898670-supplementary.pdf]

Keratoconus Risk Investigative Survey

Please tick the appropriate box for each question

* Indicates required question

1. What is your gender *

Mark only one oval.

- Male
- Female

2. What is your age? *

Mark only one oval.

- 12
- 13
- 14
- 15
- 16
- 17

3. What is your ethnicity? *

Mark only one oval.

- East Indian
- African
- Chinese
- Mixed

4. What form are you in? *

Mark only one oval.

- Form 1
- Form 2
- Form 3
- Form 4
- Form 5

5. Have you ever heard of the eye condition called Keratoconus? *

Mark only one oval.

- Yes
- No

6. How would you rate your knowledge of Keratoconus on a scale of 1 (no knowledge) to 5 (excellent knowledge)? *

Mark only one oval.

	1	2	3	4	5	
No k						Excellent knowledge

7. Has anyone in your family been diagnosed with Keratoconus? *

Mark only one oval.

- Mother
- Father
- Brother/Sister
- Cousin
- No relative

8. Are you parents blood relatives/related to each other? *

Mark only one oval.

- 1st cousins
- 2nd cousins
- Distant relatives
- Not related

9. How many hours on average per week do you spend outside in the sunlight? *

Mark only one oval.

- Less than 8 hours
- 8-24 hours
- More than 24 hours

10. How many hours on average per week do you spend doing near tasks? (reading/using cellphone/computer games) *

Mark only one oval.

- Less than 8 hours
- 8-24 hours
- More than 24 hours

11. Tick the foods that you eat at least twice a week *

Check all that apply.

- [ ] Fish/Chicken
- [ ] Red Meat
- [ ] Eggs
- [ ] Beans
- [ ] Milk
- [ ] Rice/Pap/Pasta/Bread
- [ ] Vegetables
- [ ] Fruit

12. Do you have any of the following atopic diseases/allergies

Check all that apply.

- [ ] Eczema (skin rash)
- [ ] Hayfever
- [ ] Vernal Keratoconjunctivitis
- [ ] Asthma
- [ ] Food allergies
- [ ] Pollen/dust allergies
- [ ] Animal fur allergies

13. Do you have any of the following systemic diseases?

Check all that apply.

- [ ] Down syndrome
- [ ] Osteogenesis imperfecta
- [ ] Ehlers-Danlos syndrome

14. Do you wear glasses? *

Mark only one oval.

- ( ) Yes
- ( ) No

15. If yes, how well do you see through your glasses on a scale of 1 (very blurry) to 5 (very clear)?

Mark only one oval.

	1	2	3	4	5	
Very	( )	( )	( )	( )	( )	Very Clear

16. If you do wear glasses, how often do you need to change your spectacles?

Mark only one oval.

- Every 6 months
- Every year
- Every 2 years
- Less than every 2 years
- Never changed

17. How would you rate yourself on how often you rub your eyes on a scale of 1 (never) to 5 (very often)? *

Mark only one oval.

	1	2	3	4	5	
Nev						Very Often

18. How sensitive are you to bright lights on a scale of 1 (not sensitive) to 5 (very sensitive)? *

Mark only one oval.

	1	2	3	4	5	
Not						Very sensitive

19. Do you wear/have you worn rigid contact lenses? *

Mark only one oval.

- Yes
- No

20. Have you ever had LASIK eye surgery previously? *

Mark only one oval.

- ( ) Yes
- ( ) No

This content is neither created nor endorsed by Google.

Google Forms
